# Malignancy rate of biopsied suspicious bone lesions identified on FDG PET/CT

**DOI:** 10.1007/s00259-015-3282-4

**Published:** 2016-01-04

**Authors:** Hugo J. A. Adams, John M. H. de Klerk, Ben G. F. Heggelman, Stefan V. Dubois, Thomas C. Kwee

**Affiliations:** Department of Radiology and Nuclear Medicine, University Medical Center Utrecht, Heidelberglaan 100, 3584 CX Utrecht, The Netherlands; Department of Nuclear Medicine, Meander Medical Center, Amersfoort, The Netherlands; Department of Radiology, Meander Medical Center, Amersfoort, The Netherlands; Department of Pathology, Meander Medical Center, Amersfoort, The Netherlands

**Keywords:** Biopsy, Bone, CT-guided, FDG PET/CT

## Abstract

**Purpose:**

To determine the malignancy rate of bone lesions identified on FDG PET/CT in patients who have undergone CT-guided biopsy because of the suspicion of malignancy.

**Methods:**

This single-centre retrospective study spanned eight consecutive years and included all patients who underwent both FDG PET/CT and CT-guided bone biopsy because of the suspicion of malignancy. The positive predictive value (PPV) for malignancy was calculated, and different patient and imaging characteristics were compared between malignant and benign bone lesions.

**Results:**

Of 102 included patients with bone lesions that all showed FDG uptake exceeding mediastinal uptake, bone biopsy showed a malignant lesion in 91 patients, yielding a PPV for malignancy of 89.2 % (95 % CI 81.7 – 93.9 %). In the 94 patients with bone lesions that showed FDG uptake exceeding liver uptake, bone biopsy showed a malignant lesion in 83 patients, yielding a PPV for malignancy of 88.3 % (95 % CI 80.1 – 93.5 %). Higher age, bone marrow replacement of the lesion seen on CT, expansion of the lesion seen on CT, and presence of multifocal lesions on FDG PET/CT were significantly more frequent in patients with malignant lesions than in those with benign bone lesions (*P* = 0.044, *P* = 0.009, *P* = 0.015, and *P* = 0.019, respectively). Furthermore, there was a trend towards a higher incidence of cortical destruction (*P* = 0.056) and surrounding soft tissue mass (*P* = 0.063) in patients with malignant bone lesions.

**Conclusion:**

The PPV for malignancy of suspicious bone lesions identified on FDG PET/CT is not sufficiently high to justify changes in patient management without histopathological confirmation. Nevertheless, ancillary patient and imaging characteristics may increase the likelihood of a malignant bone lesion.

## Introduction

Bone metastases are a frequent complication of cancer, occurring in up to 70 % of patients with advanced breast or prostate cancer, and in approximately 15 – 30 % of patients with carcinoma of the lung, colon, stomach, bladder, uterus, rectum, thyroid or kidney [1]. Timely and accurate detection of bone metastatic disease is important for treatment planning and prognostication. ^18^F-FDG PET/CT is currently regarded as the most accurate cross-sectional whole-body imaging modality for the evaluation of many cancers, and is increasingly being used for this purpose in clinical practice. The addition of functional FDG PET information to anatomical CT findings may improve the detection of malignant bone disease [2]. Nevertheless, histopathological examination remains the method of choice for the final diagnosis of bone lesions that are suspicious for malignancy. Biopsy under CT guidance plays an important role in obtaining tissue for histopathological examination of suspicious bone lesions identified on FDG PET/CT. Although percutaneous CT-guided bone biopsy is generally regarded as a safe and effective procedure, it is still burdensome to the patient, not completely risk-free and costly, and there is a delay in diagnosis when the acquired specimen has to be decalcified before it can be histologically examined [3–5]. In addition, not every patient may be a candidate for CT-guided biopsy due to physical or mental health issues.

 In this context, it would be of interest to know what proportion of suspicious bone lesions identified on FDG PET/CT prove to be malignant on biopsy. Although it can be argued that FDG uptake is not specific for malignancy, the diagnostic yield of CT-guided biopsy of focal FDG-avid (i.e. FDG uptake exceeding liver FDG uptake) bone lesions is still unknown. Previous studies related to this topic lacked histopathological correlation [6, 7] or suffered from a small sample size [8]. If FDG PET/CT achieves a high positive predictive value (PPV), the need for bone biopsy in every patient may be reconsidered. The purpose of this study was therefore to determine the malignancy rate of bone lesions identified on FDG PET/CT who have undergone CT-guided biopsy because of the suspicion of malignancy.

## Materials and methods

### Study design and patients

This retrospective study was approved by the local institutional review board and the requirement for written informed consent was waived. The Picture Archiving and Communication System (PACS) of the Meander Medical Center was searched for all patients who had undergone both FDG PET/CT and CT-guided biopsy between September 2007 and September 2015, and their records were reviewed to determine age, gender, presence of bone pain, and reported weight loss. The start date of September 2007 was chosen because this was when integrated PET/CT replaced stand-alone PET at our institution. The Meander Medical Center is a large university-affiliated general teaching hospital centrally located in The Netherlands. It provides care to patients with almost all types of cancer, with ample experience in the treatment of breast, prostate, lung, colorectal, haematological, renal, ovarian and gastric malignancies. However, patients with suspicion of a primary malignant bone tumour are referred directly to another institution with expertise in orthopaedic oncology for diagnosis and treatment, and these patients were therefore not included in this study.

 Inclusion criteria for this study were: availability of a CT-guided bone biopsy that was performed because of the suspicion of malignancy and availability of a FDG PET/CT scan within 1 month of CT-guided biopsy. Exclusion criteria were: patients who underwent CT-guided bone biopsy because of (suspicion of) infection or inflammation, therapy (either radiation therapy or chemotherapy) between bone biopsy and the FDG PET/CT scan, technically failed bone biopsy (i.e. no bone tissue acquired), no clear histopathological diagnosis, and no or insufficient tissue for histopathological diagnosis as determined by the attending pathologist.

### FDG PET/CT

All patients underwent integrated FDG PET/CT. Between September 2007 and December 2013 a Biograph 40 TruePoint system (Siemens Healthcare, Erlangen, Germany) was used, and between January 2014 and September 2015 a Biograph TruePoint TrueV system (Siemens) was used for FDG PET/CT scanning. Patients fasted for at least 6 h and blood glucose levels were checked to ensure that they were less than 11 mmol/L before intravenous injection of FDG ([3 × weight in kilograms] megabecquerels for the Biograph 40 TruePoint and [0.0209 × weight in kilograms^2,047^] megabecquerels for the Biograph TruePoint TrueV). Approximately 60 min after FDG administration, a 3D PET acquisition was initiated from the mid-thigh to the skull base, in five to seven bed positions (with 3 min per bed position). A low-dose CT scan was used for transmission scanning, and the PET images were reconstructed using a weighted iterative ordered-subsets expectation maximization algorithm. A contrast-enhanced full-dose CT scan in the portal venous phase was performed as part of the FDG PET/CT examination in 61 of 102 included patients (59.8 %). In addition, all patients underwent a full-dose CT scan in a separate session for CT-guided bone biopsy planning.

### FDG PET/CT evaluation

Using a PACS workstation (IMPAX 6.6.1.1527; AGFA, Mortsel, Belgium), all FDG PET/CT images were evaluated by an experienced reader (T.C.K.) who was blinded to the clinical, laboratory, pathological and follow-up findings. However, the reader was informed about the location of each bone lesion for which CT-guided biopsy was performed. The FDG uptake of each bone lesion was visually assessed using a five-point grading scale: *1* no uptake, *2* uptake lower than or the same as the mediastinum, *3* uptake higher than the mediastinum but lower than or the same as the liver, *4* uptake moderately higher than the liver, and *5* uptake markedly higher than the liver. FDG PET images were analysed in the axial plane with a slice thickness of 5 mm and in maximum intensity projection display for this purpose. Furthermore, each bone lesion was assessed for the presence or absence of lysis, sclerosis, cortical destruction, bone marrow replacement, surrounding soft-tissue mass, expansiveness, and vertebral collapse (for a vertebral lesion). To that end, full-dose CT images (either from the FDG PET/CT examination or the CT-guided bone biopsy planning) were analysed in different planes (axial, sagittal and coronal) with the slice thickness in the range 2 – 5 mm. Additionally, the number of bone lesions in each patient was dichotomized as one or multiple (i.e. more than one), as determined on FDG PET/CT.

### CT-guided bone biopsy

CT-guided biopsies were performed as part of routine clinical care by or under the supervision of one of the attending radiologists with at least 3 years experience with this procedure. All patients underwent core needle biopsy. The core needle size ranged between 9 and 18 gauge, depending on the preference of the attending radiologist as determined for each patient individually.

### Histopathological examination

The bone biopsy specimens obtained were first decalcified (when needed) and then evaluated as part of routine clinical care by one of the attending pathologists.

### Diagnostic analysis

The PPV for malignancy (i.e. number of malignant lesions/number of all malignant and benign lesions) was calculated for all biopsied lesions and for lesions with FDG uptake exceeding liver uptake separately, along with 95 % confidence intervals (CI). Furthermore, the following factors were compared between malignant and benign lesions, using the Fisher's exact test for binary data and the Mann-Whitney *U* test for non-Gaussian continuous and ordinal data:

- Patient age: continuous variable
- Gender: male vs. female
- Reported bone pain: presence vs. absence
- Reported weight loss: presence vs. absence
- Lesional FDG avidity: according to the five-point grading scale
- CT abnormalities: presence vs. absence of any lesional CT abnormality
- Specific lesional CT characteristics: presence vs. absence of lysis, sclerosis, cortical destruction, bone marrow replacement (this item only scored as positive if a hyperattenuating soft-tissue mass was clearly visible in the bone marrow space), surrounding soft-tissue mass, expansiveness and vertebral collapse
- Number of bone lesions: one vs. multiple

All tests were two-sided and *P* values less than 0.05 were considered statistically significant. Statistical analyses were performed using SPSS version 17.0.0 (SPSS Inc., Chicago, IL).

## Results

### Patients

Between September 2007 and September 2015, 225 patients underwent a CT-guided bone biopsy. Of these 225 patients, 83 were excluded because no FDG PET/CT scan was performed within 1 month of the bone biopsy, 31 were excluded because of (suspicion of) spondylodiscitis, 4 were excluded because of (suspicion of) sacroiliitis, 2 were excluded because there was no or insufficient tissue for histopathological diagnosis as determined by the attending pathologist, 1 was excluded because the biopsy needle fractured during the procedure as a result of which no bone tissue was acquired, 1 was excluded because of radiation therapy between the FDG PET/CT scan and the bone biopsy, and 1 was excluded because no clear histopathological diagnosis could be made. Thus, 102 patients (63 men, 39 women, mean age 63.3 years, range 19 – 86 years) were finally included in this study. The basic characteristics of the included patients are shown in Table 1. The bone lesions were located in the iliac bone (43 patients), vertebrae (22), sacrum (9), ribs (9), femur (6), scapula (5), sternum (4), humerus (3) and clavicle (1). All lesions that were biopsied had an FDG avidity score of 3 – 5 according to the five-point scale. Three patients underwent repeat biopsy because the first biopsy was inconclusive.

**Table 1:** Basic characteristics of included patients

Characteristic	Value
Patients, *n*	102
Male/female	63/39
Diagnosis of malignant disease, *n* (%)	91 (89.2)
Age (years)	
Mean	63.3
Median	67.0
Range	19 – 86
Patients receiving chemotherapy during the 3 months before FDG PET/CT and CT-guided bone biopsy, *n* (%)	10 (9.8)
Time between FDG PET/CT and bone biopsy (days)	
Mean	8.3
Median	7.0
Range	0 – 31
Patients undergoing FDG PET/CT before CT-guided bone biopsy, *n* (%)	92 (90.2)

### Diagnostic yield

**Fig. 1:**
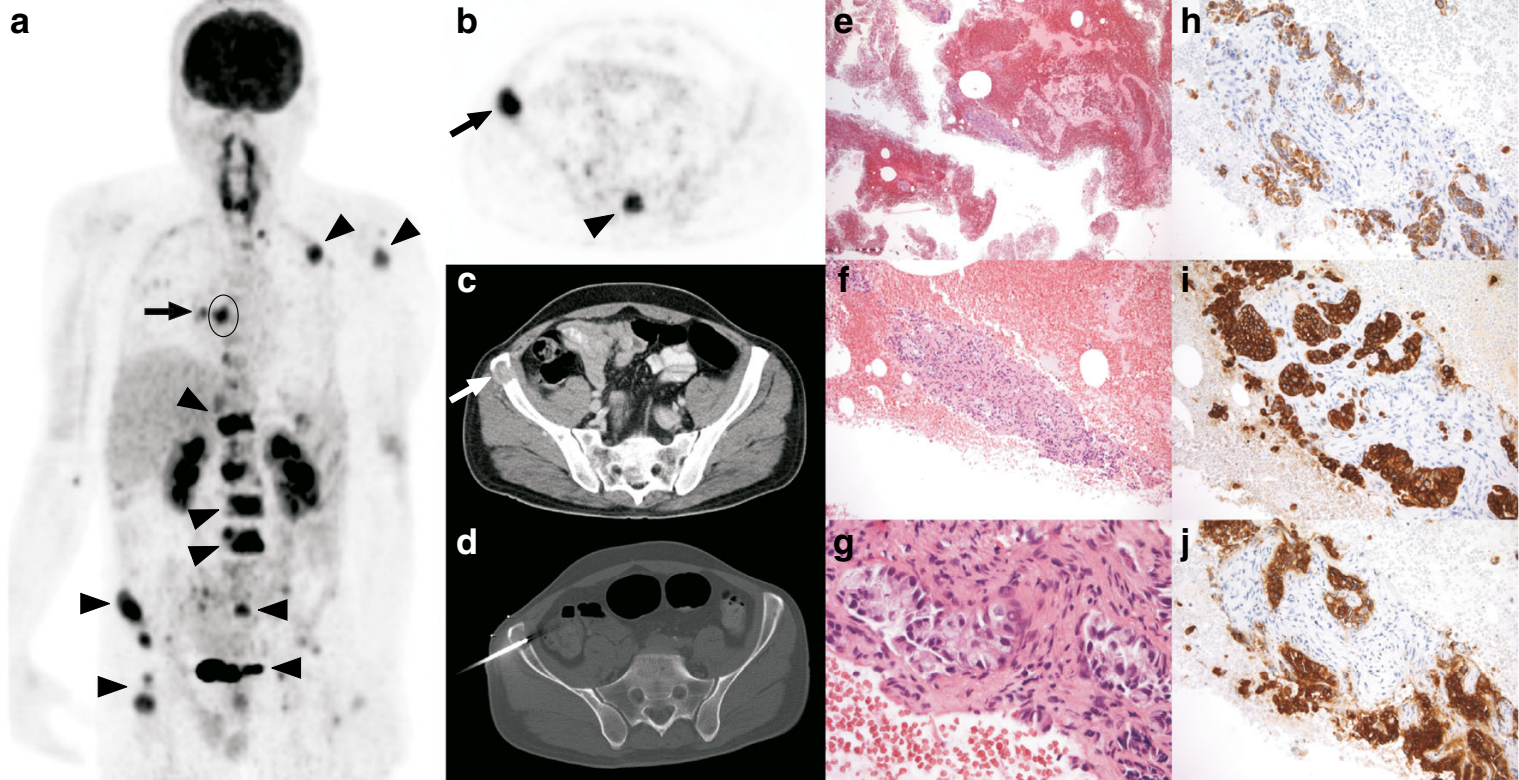
A 46-year-old man presented with severe back pain. MRI of the lumbar spine (not shown) demonstrated multiple vertebral bone lesions, suspicious for bone metastatic disease. **a** The coronal maximum intensity projection FDG PET image shows multiple FDG-avid bone lesions in the left proximal humerus, left-sided ribs, multiple vertebrae, sacrum, iliac wings, and right proximal femur (*arrowheads*). Also note an FDG-avid lesion in the right lower lobe (*arrow*) with ipsilateral mediastinal lymphadenopathy (*encircled*). **b**, **c** The axial FDG PET image (**b**) and the corresponding CT image with soft tissue settings (**c**) at the level of the pelvis show FDG-avid lesions in the sacrum (*arrowhead*) and right anterior iliac crest (*arrows*), the latter with bone marrow replacement and expansiveness, features that were found to be significantly more frequent in malignant than in benign lesions. **d** The right anterior iliac crest lesion was biopsied under CT guidance. **e–j** Histopathological specimens (**e–g** H&E staining, **h–j** immunohistochemical staining): **e** several fragments of tissue in a background of fresh blood, but no bone (original magnification ×25); **f** larger fragment with several epithelial structures and a desmoplastic background (original magnification ×100); **g** detail shows clusters and tubules of medium large epithelioid cells with partly amphophilic cytoplasm and mostly hyperchromatic, polygonal atypical nuclei, set in a dense desmoplastic stroma, consistent with (metastatic) adenocarcinoma (original magnification ×400); **h–j** the adenocarcinoma stains positive for the broad spectrum cytokeratin MNF116 (**h** original magnification ×200), for cytokeratin 7 (**i**), and for CEA (**j**), but CK20, cdx2, TTF-1, p501s and PSA were negative (not shown). Despite the negative TTF-1, the diagnosis of metastatic lung adenocarcinoma could be established

**Fig. 2:**
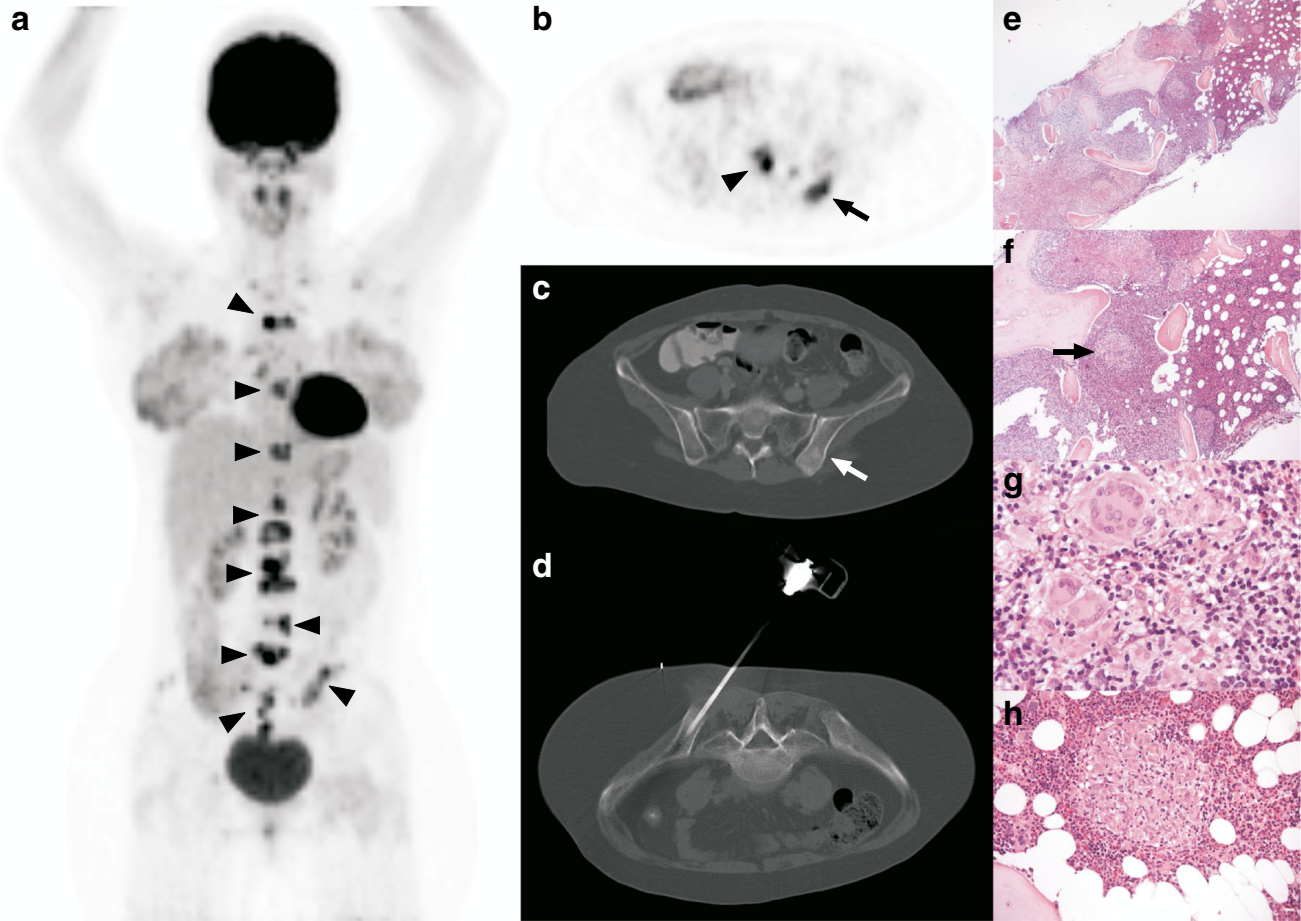
A 33-year-old woman presented with severe lower back pain. MRI of the lumbar spine (not shown) demonstrated multiple vertebral bone lesions, suspicious for bone metastatic disease. **a** The coronal maximum intensity projection FDG PET image shows multiple FDG-avid foci in the sternum, thoracic and lumbosacral spine, and both iliac wings (*arrowheads*). **b**, **c** The axial FDG PET image (**b**) and the corresponding CT image with bone window settings (**c**) at the level of the pelvis show FDG-avid lesions in the sacrum (*arrowhead*) and left posterior iliac crest (*arrows*), the latter with a sclerotic appearance. **d** The left posterior iliac crest lesion was biopsied under CT guidance. **e–h** Histopathological specimens (H&E staining): **e** the centre of the bone biopsy shows the transition between pre-existing haematopoietic bone marrow in the upper right third and extensive inflammatory infiltrate in the lower left two thirds; the diameter of the bony trabeculae (*light pink*) varies, and the layering of the trabeculae can be discerned (original magnification ×25); **f** enlargement shows several areas of necrosis, most prominent in the upper left, and a cluster of multinucleated giant cells (*arrow*) (original magnification ×50). **g** further enlargement of the same area (original magnification ×400); **h** sharply delineated granuloma surrounded by pre-existing haematopoietic bone marrow is also demonstrated (original magnification ×200). Ziehl-Neelsen staining for acid-fast bacillae on the biopsy was negative (not shown), but the Mantoux skin test was strongly positive with blistering. Therefore, the diagnosis of bone tuberculosis could be established

**Fig. 3:**
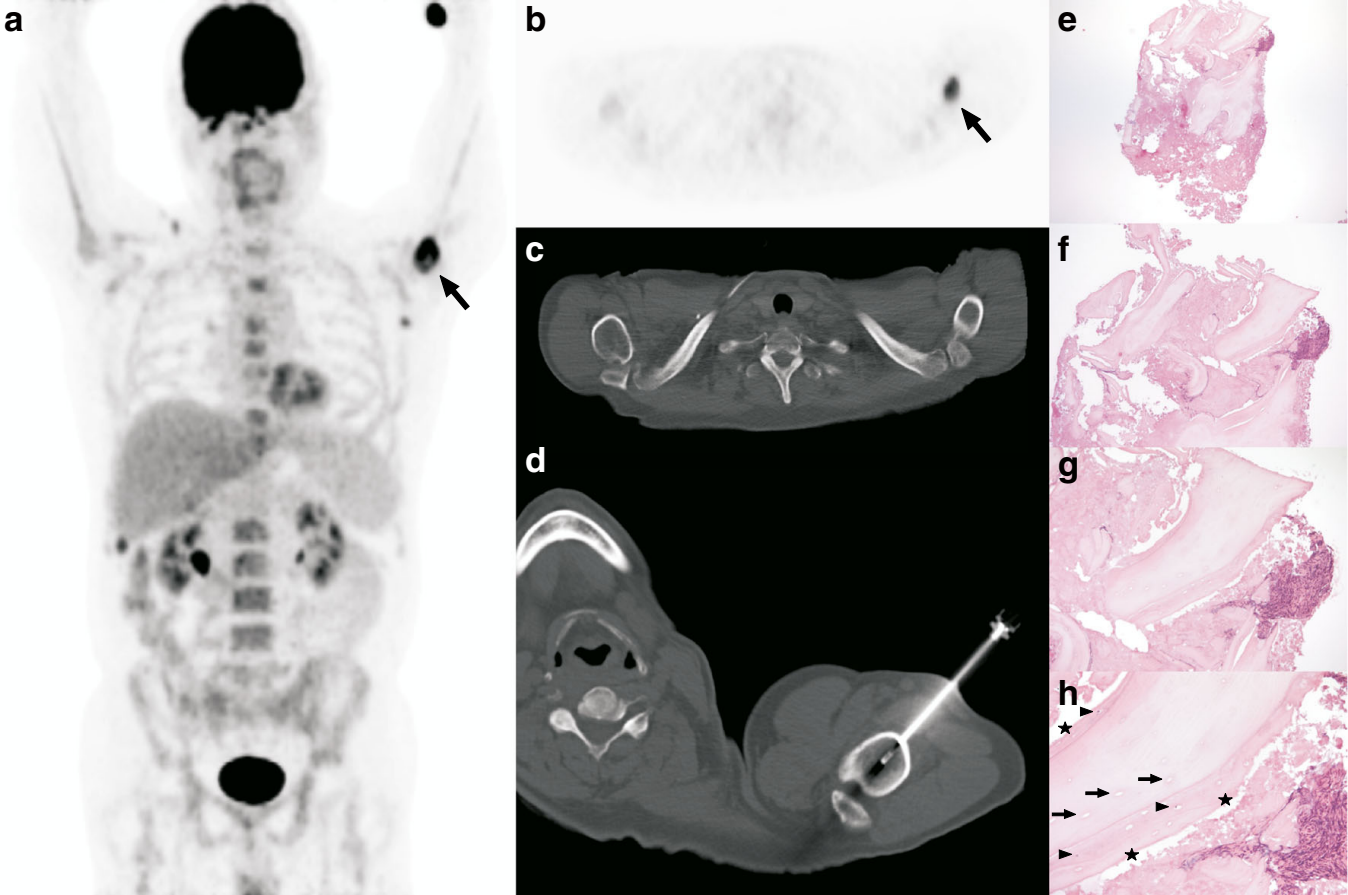
A 58-year-old man who was treated with DHAP-VIM-DHAP (DHAP, cisplatin-cytarabine-dexamethasone; VIM, etoposide-ifosfamide-methotrexate) before autologous stem cell transplantation because of relapsed peripheral T-cell non-Hodgkin lymphoma. **a** The coronal maximum intensity projection FDG PET image shows a new FDG-avid lesion in the left proximal humerus (*arrow*) that developed during chemotherapy. **b**, **c** The axial FDG PET image (**b**) and the corresponding CT image with bone window settings (**c**) at the level of the left proximal humerus show the FDG-avid lesion (*arrow*) without any clear abnormalities seen on CT. **d** The lesion was biopsied under CT guidance. **e–h** Histopathological specimens (H&E staining): **e** one of the larger fragments of the biopsy of the left proximal humerus shows irregular and enlarged, clearly layered bony trabeculae (original magnification ×25); the darker portion in the lower area consists of amorphous eosinophilic material, partially consisting of destroyed bone; the upper right area shows smeared cells, of which the provenance cannot be ascertained; **f**, **g** enlargement (**f** original magnification ×50) and further enlargement (**g** original magnification ×100) show the apposed rim of “new” bone; **h** yet further enlargement (original magnification ×200) clearly shows the apposed rim of “new” bone, with the central region of necrotic bone showing empty lacunae (*arrows*); in the surrounding apposed lamellar bone a few nuclei (*arrowheads*) can be seen, which could fit with (partial) regeneration after avascular necrosis; however, new woven bone as well as a rim of osteoblasts at the periphery are missing (*stars*). Based on these histopathological findings, avascular necrosis is the most likely diagnosis

Of the 102 patients with bone lesions that all showed FDG uptake exceeding mediastinal uptake, bone biopsy showed a malignant lesion in 91 and a benign lesion in 11, which yielding a PPV for malignancy of 89.2 % (95 % CI 81.7 – 93.9 %). Table 2 shows the types and numbers of malignant and benign bone lesions diagnosed. Of note, two patients with a known cancer (prostate cancer in both) were diagnosed with another cancer based on the bone biopsy (lung carcinoma metastasis in both). Of the 94 patients with bone lesions with FDG uptake exceeding liver uptake, bone biopsy showed a malignant lesion in 83 and a benign lesion in 11, which yielding a PPV for malignancy of 88.3 % (95 % CI 80.1 – 93.5 %). Higher age, bone marrow replacement of the lesion on the CT scan, expansion of the lesion on the CT scan, and presence of multifocal lesions on the FDG PET/CT scan were significantly more frequent in patients with malignant than in those with benign bone lesions (*P* = 0.044, *P* = 0.009, *P* = 0.015, and *P* = 0.019, respectively). Other patient and imaging characteristics were not found to be significantly different between patients with malignant and those with benign lesions, although there was a trend towards more frequent cortical destruction (*P* = 0.056) and surrounding soft -issue mass (*P* = 0.063) in malignant bone lesions (Table 3). Representative examples are shown in Figs. 1, 2, and 3.

**Table 2:** Types and numbers of malignant and benign bone lesions diagnosed

Lesions diagnosed on bone biopsy	No. of lesions
Malignant diseases	
Lung carcinoma metastasis	44
Breast carcinoma metastasis	11
Myeloma	11
Lymphoma	8
Unspecified carcinoma metastasis	6
Gastrointestinal carcinoma metastasis	4
Renal cell carcinoma metastasis	3
Melanoma metastasis	1
Urinary tract carcinoma metastasis	1
Leiomyosarcoma metastasis	1
Thyroid carcinoma metastasis	1
Benign diseases	
Tuberculosis	2
Inflammatory infiltrate of unknown origin	2
Focal red marrow hyperplasia	1
Reactive acetabular bone marrow with multiple macrophages and foreign particles in patient with total hip prosthesis (and known lung carcinoma)	1
Avascular necrosis	1
Osteoporotic fracture	1
Osteomyelitis	1
Giant cell granuloma	1
Gout	1

**Table 3:** Patient and imaging characteristics in patients with malignant and benign bone lesions and corresponding positive and negative predictive values for malignancy

Characteristic		Malignant	Benign	*P* value (malignant vs. benign)	Positive predictive value	Negative predictive value
Age (years), median		67.0^a^	52.0	0.044^b^	47/51 (92.2 %)^d^	7/51 (13.7 %)^d^
Male sex		58/91 (63.7 %)	5/11 (45.5 %)	0.326^c^	58/63 (92.1 %)	6/39 (15.4 %)
Bone pain		60/91 (65.9 %)	6/11 (54.5 %)	0.512^c^	60/66 (90.9 %)	5/36 (13.9 %)
Weight loss		25/91 (27.5 %)	4/11 (36.4 %)	0.503^c^	25/29 (86.2 %)	7/73 (9.6 %)
CT abnormal		77/91 (84.6 %)	7/11 (63.6 %)	0.101^c^	77/84 (91.7 % )	4/18 (22.2 %)
CT lytic		67/91 (73.6 %)	5/11 (45.5 %)	0.078^c^	67/72 (93.1 %)	6/30 (20 %)
CT sclerotic		30/91 (33.0 %)	3/11 (27.3 %)	1.000^c^	30/33 (90.9 %)	8/69 (11.6 %)
CT cortical destruction		54/91 (59.3 %)	3/11 (27.3 %)	0.056^c^	54/57 (94.7 %)	8/45 (17.8 %)
CT marrow replacement		47/91 (51.6 %)	1/11 (9.1 %)	0.009^c^	47/48 (97.9 %)	10/54 (18.5 %)
CT surrounding soft tissue mass		24/91 (26.4 %)	0/11 (0.0 %)	0.063^c^	24/24 (100.0 %)	11/78 (14.1 %)
CT expansive		33/91 (36.3 %)	0/11 (0.0 %)	0.015^c^	33/33 (100.0 %)	11/69 (15.9 %)
CT vertebral deformity		8/91 (8.8 %)	1/11 (9.1 %)	1.000^c^	8/9 (88.9 %)	10/93 (10.8 %)
FDG avidity (five-point scale)	3	8 (8.8 %)	0 (0.0 %)	0.096^b^	–^e^	
	4	11 (12.1 %)	0 (0.0 %)			
	5	72 (79.1 %)	11 (100.0 %)			
Multifocal bone lesions on FDG PET/CT		73/91 (80.2 %)	5/11 (45.5 %)	0.019^c^	73/78 (93.6 %)	6/24 (25.0 %)

^a^Not normally distributed according to Kolmogorov-Smirnov test (*P* < 0.001)

^b^Mann-Whitney *U* test

^c^Fisher’s exact test

^d^Age dichotomized using the median

^e^Analysis not performed because FDG avidity score could not be dichotomized

## Discussion

The results of this study show that although the vast majority of bone lesions identified on FDG PET/CT that are referred for CT-guided bone biopsy because of the suspicion of malignancy are indeed malignant, there is still a non-negligible number of bone lesions that prove to be benign. Importantly, this notion also applies to bone lesions that are highly FDG-avid, i.e. with FDG uptake exceeding liver uptake. Thus, FDG PET/CT appears to be insufficiently specific to (partially) replace CT-guided bone biopsy to confirm malignancy. Interestingly, higher age, bone marrow replacement of the lesion seen on CT, expansion of the lesion seen on CT, and presence of multifocal lesions on FDG PET/CT were significantly more frequent in malignant than in benign bone lesions. There was also trend towards a significantly higher frequency of cortical destruction and surrounding soft tissue mass in malignant bone lesions. This information may be helpful to determine the most likely imaging-based diagnosis if bone biopsy cannot be performed for whatever reason.

A limited number of previous studies on this topic have been performed. Toomayan and Major [9] retrospectively reviewed all CT-guided bone biopsies performed at their institution over a period of 3 years in patients with a history of a single biopsy-proven malignancy. Of 93 included patients, 82 (88 %) showed skeletal involvement by the known malignancy, 7 showed a new malignancy, and 4 (4 %) showed no malignancy. Thus biopsy of a suspicious skeletal lesion in a patients with a solitary known malignancy revealed a new malignancy or no evidence of malignancy in 12 % of patients, emphasizing the importance of biopsy. However, Toomayan and Major [9] did not assess the role of FDG PET/CT in this setting.

 In another study, Taira et al. [7] retrospectively reviewed the FDG PET/CT reports in 59 patients with a histopathologically proven malignancy, who had a total of 113 bone lesions. Of 47 bone lesions with positive findings on both FDG PET and CT, 46 were malignant and 1 was benign, yielding a PPV of 97.9 % (95 % CI 88.9 – 99.6 %). Of 31 lesions with positive findings on FDG PET and negative findings on CT, 19 were malignant and 12 were benign, yielding a PPV of 61.3 % (95 % CI 43.8 – 76.3 %). Of 35 lesions with negative findings on FDG PET and positive findings on CT, 6 were malignant and 29 were benign, yielding a PPV of 17.1 % (95 % CI 8.1 – 32.7 %). Independently, the PPV of FDG PET for all lesions with positive findings was significantly higher than that of CT for all lesions with positive findings. The chemotherapy status for lesions with positive findings on CT and the number of lesions per patient was found to significantly associated with the PPV of the examinations (*P* = 0.02 and *P* < 0.001, respectively). Taira et al. [7] concluded that CT has a very high PPV for bone metastases (98 %) when the findings on FDG PET and CT are concordant; however, in lesions with discordant FDG PET and CT findings in the integrated examination, PPV is markedly diminished. Major disadvantages of the study by Taira et al. [7], however, are the lack of a histopathological reference standard in the vast majority of cases (only 13 of 113 lesions were histopathologically assessed), and the lack of standardized FDG PET and CT criteria. Therefore, the validity and applicability of their results are questionable.

 In yet another study, Nguyen et al. [8] retrospectively searched their radiology procedure database for all FDG PET-positive lesions that were biopsied under imaging-guidance. Of 26 biopsied bone lesions, 25 proved to be malignant, yielding a PPV of 96.2 % (95 % CI 81.1 – 99.3 %). However, the study by Nguyen et al. [8] was limited by a small sample size and also lacked a clear definition of FDG PET positivity. Unlike previous research, the present study included the largest sample size of patients who had all undergone both FDG PET/CT (which was interpreted with standardized criteria) and CT-guided bone biopsy followed by histopathological examination, which allowed more solid conclusions to be drawn on the roles of these diagnostic procedures with respect to the assessment of (suspicious) bone lesions.

This study had several limitations. First, the study was performed in a large hospital centrally located in The Netherlands, as a result of which the results are only applicable to a similar (Western) population. In non-Western countries, the PPV for malignancy of suspicious biopsied FDG-avid bone lesions may be lower, for example due to the higher prevalence of (bone) tuberculosis in such countries. Furthermore, the results are not applicable to primary bone tumours, because these were not included in the present study. In addition, no paediatric patients were included. Second, due to the retrospective nature of this study, only patients who underwent FDG PET/CT and who were referred for CT-guided biopsy were included, which may have introduced both selection and verification bias. Third, 10 of 102 patients received chemotherapy during the 3 months before FDG PET/CT and CT-guided bone biopsy (but not between the two procedures), which may have affected FDG uptake in bone. However, this reflects clinical practice, and all of the biopsied bone lesions in these patients were still FDG-avid (i.e. FDG uptake score of 3 in two patients, 4 in one patients, and 5 in seven patients). Fourth, 10 of 102 (9.8 %) patients underwent CT-guided bone biopsy before FDG PET/CT, and this may have affected the FDG PET/CT findings. Fifth, no (semi)quantitative FDG uptake measurements were performed, because two different PET/CT systems were used during the period of the study, and it is known that this can significantly affect standardized uptake value measurements [10]. Sixth, all FDG PET/CT scans were performed approximately 60 min after FDG injection. Dual time-point FDG PET has been reported to be potentially helpful in the differentiation of malignant tumours from benign ones [11] but this was outside the scope of this study. Seventh, not all patients had undergone a contrast-enhanced CT scan. However, it is unlikely that this affected the CT-based assessment of bone lesions, because all patients had an available full-dose CT scan.

In conclusion, the PPV for malignancy of suspicious bone lesions identified on FDG PET/CT is not sufficiently high to justify changes in patient management without histopathological confirmation. Nevertheless, ancillary patient and imaging characteristics may help to quantify the likelihood of a malignant bone lesion.
